# Acute appendicitis revealing a giant mesenteric pseudocyst: case report

**DOI:** 10.11604/pamj.2022.41.178.32577

**Published:** 2022-03-04

**Authors:** Salsabil Mohamed Sabounji, Mbaye Fall, Doudou Gueye, Cheikh Seye, Gabriel Ngom

**Affiliations:** 1 Cheikh Anta Diop University, Dakar, Senegal,; 2 Pediatric Surgery Department, Heinrich Lübke Regional Hospital, Diourbel, Senegal,; 3 Pediatric Surgery Department, Aristide Le Dantec Hospital, Dakar, Senegal,; 4 Pediatric Surgery Department, Albert Royer Children´s Hospital, Dakar, Senegal,; 5 Alioune Diop University of Bambey, Diourbel, Senegal

**Keywords:** Pseudo-mesenteric, mesenteric cyst, giant cyst, child, case report

## Abstract

Mesenteric pseuodycst is a very rare benign childhood tumor, accounting for less than 1 out of 250,000 hospital admissions. We here report a case of giant mesenteric pseudocyst incidentally detected in a 11-year-old boy with acute appendicitis. He complained of persistent abdominal pain for the past 48 hours. He had a history of intermittent pain for several months. Physical examination showed fever and abdominal pain. Ultrasonography showed large peritoneal fluid related to peritonitis probably of appendicular origin. The patient underwent exploratory laparotomy revealing giant abdominal mesenteric cyst and acute appendicitis. Open resection of the cyst and appendectomy were performed. The diagnosis of uncomplicated acute appendicitis associated with mesenteric pseudocyst was made. Preoperative diagnosis of pseudomesenteric cysts is a clinical challenge. Knowledge is essential and suspicion should be maintained in patients with nonspecific symptoms.

## Introduction

Mesenteric cyst is a rare benign tumor. It can be located anywhere in the mesentery from the duodenum to the rectum [1]. More than half of the cysts are found in the small bowel mesentery, and, in particular, in the ileum [2]. The incidence has been estimated to be almost 1 in every 250,000 hospital admissions [1]. In pediatric patients, few reports have stated their incidence between 1/10000 and 1/25000 [3]. Their precise origin is still unclear but it´s likely thought that the cause of mesenteric cysts is a benign ectopic lymphatic proliferation in the mesentery that are not related to other parts of the mesentery [4,5]. The diagnosis of mesenteric cysts is a clinical challenge due to nonspecific symptoms. We report a case of a giant mesenteric cyst incidentally detected in a child with appendicitis.

## Patient and observation

**Patient information:** an 11-year-old boy was admitted to the emergency department with persistent abdominal pain for 48hours with vomiting. The patient reported a history of intermittent pain with no specific symptoms for several months. No other relevant history was noted.

**Clinical findings:** on admission, the patient had a fever (38.5°C). Abdominal examination showed a slight epigastric abdominal swelling, abdominal tenderness and pain in the right iliac fossa and epigastric region.

**Diagnostic assessment:** blood tests revealed normal leukocytes count and high levels of inflammatory markers, but no other relevant findings. Abdominal ultrasound showed large peritoneal effusion related to peritonitis of probably appendicular origin. For further assessment, a computed tomography (CT) scan was advised but could not be done due to limited access to emergency care services. The diagnostic hypothesis was complicated appendicitis.

**Figure 1:**
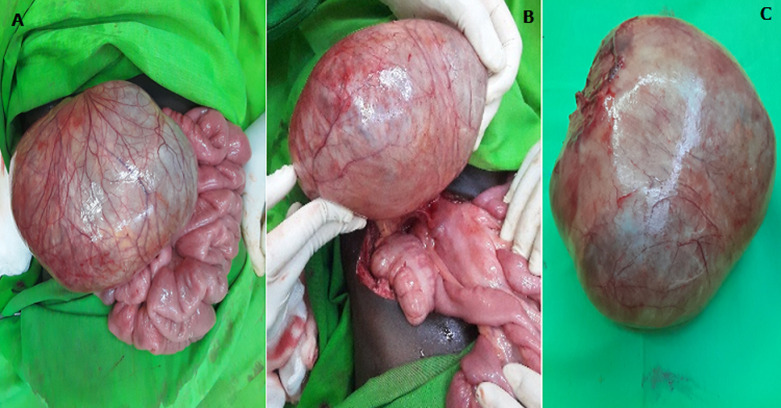
intraoperative images showing: A) resected specimen of mesenteric cyst; B aspect of the posterior wall of the cyst connected with the root of the mesentery; C) mesentery cyst after the skin section

**Figure 2:**
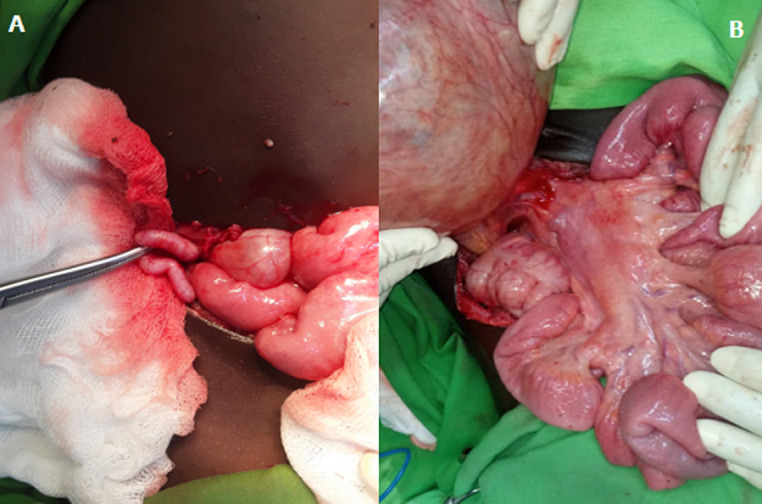
aspect of acute appendicitis (A); multiples lymph nodes of different size (B)

**Therapeutic intervention:** after resuscitation, an exploratory laparotomy was performed. It revealed a giant abdominal unilocular cyst adherent to the mesenteric wall of the small bowel measuring 17cmx12cm and acute appendicitis. We noticed multiple inflamed lymph nodes of different sizes (Figure 1). The patient underwent open resection of the cyst and appendectomy. Appropriate specimens were sent for the histopathological exams (Figure 2). After 5 days, the patient was discharged.

**Follow-up and outcome of interventions:** the postoperative course was uneventful and there were no significant symptoms and no clinical evidence of recurrence during the 3-month follow-up. Histological examination confirmed the diagnosis of the mesenteric pseudocyst, with inflammatory infiltrate. Fluid cytology was negative for malignancy. The diagnosis of uncomplicated acute appendicitis with mesenteric lymphadenitis was made.

**Informed consent:** written informed consent was obtained from the patient and his parents for publication of this case report and accompanying images. A copy of the written consent is available for review by the Editor-in-Chief of this journal on request.

## Discussion

Mesenteric pseuodycst is a rare type of mesenteric cyst, accounting for less than 1 out of 250,000 hospital admissions [6]. More than half of the cysts are found in the small bowel mesentery and, in particular, in the ileum [2]. Mesenteric cysts usually have one of five pathologic patterns: lymphangioma, enteric duplication, enteric cysts, and mesothelial or nonpancreatic pseudocysts [7]. An updated features-based histopathological classification was recently suggested: 1) cysts of lymphatic origin (simple lymphatic cyst and lymphangioma), (2) cysts of mesothelial origin (simple mesothelial cyst, benign cystic mesothelioma and malignant cystic mesothelioma), (3) cysts of enteric origin (enteric cyst and enteric duplication cyst), (4) cysts of urogenital origin, (5) mature cystic teratoma (dermoid cysts), and (6) pseudocysts (infectious and traumatic cysts). Our case would likely be a pseudocyst of infectious aetiology based on associated mesenteric adenolymphitis and inflammatory infiltrate in histology associated with no history of trauma. [8].

Clinical presentation includes nonspecific abdominal pain, abdominal distention, change in bowel habit, nausea, vomiting and an abdominal mass. Physical examination is often unremarkable. Complications such as intestinal obstruction, ischaemic bowel, volvulus and peritonitis or haemorrhagic shock secondary to rupture or bleeding into the cyst, rarely occur [1]. In our patient, a history of intermittent abdominal pain was the only symptom. In this case, acute abdomen (appendicitis) incidentally revealed a mesenteric cyst, no complication occurred. Although there´s no specific sign, ultrasonography (USG) and CT scan of the abdomen can help to confirm the presence of mesenteric cyst [4,9]. In our case, USG was not contributive and limited access to CT scans was a diagnostic challenge. The standard treatment for mesenteric cysts is complete resection. This could be done either laparoscopically or through a laparotomy. Partial excision is associated with recurrence and morbidity [1,10].

## Conclusion

Mesenteric cysts are very rare benign tumors. Preoperative diagnosis is a challenge due to nonspecific symptoms. In some cases, complications could occur with patent clinical presentation. Abdominal imaging is necessary for preoperative diagnosis. Knowledge is essential, and suspicion should be maintained. Complete resection is the gold standard treatment.
